# The Reduction of Peripheral Blood CD4^+^ T Cell Indicates Persistent Organ Failure in Acute Pancreatitis

**DOI:** 10.1371/journal.pone.0125529

**Published:** 2015-05-04

**Authors:** Zhiyong Yang, Yushun Zhang, Liming Dong, Chong Yang, Shanmiao Gou, Tao Yin, Heshui Wu, Chunyou Wang

**Affiliations:** Pancreatic Disease Institute, Union Hospital, Tongji Medical College, Huazhong University of Science and Technology, Wuhan, Hubei Province, People’s Republic of China; University of Szeged, HUNGARY

## Abstract

**Objective:**

Few data are available on the potential role of inflammatory mediators and T lymphocytes in persistent organ failure (POF) in acute pancreatitis (AP). We conducted a retrospective study to characterize their role in the progression of POF in AP.

**Methods:**

A total of 69 AP patients presented within 24 hours from symptom onset developing organ failure (OF) on admission were included in our study. There were 39 patients suffering from POF and 30 from transient OF (TOF). On the 1st, 3rd and 7th days after admission, blood samples were collected for biochemical concentration monitoring including serum IL-1β, IL-6, TNF-α and high-sensitivity C-reactive protein (hs-CRP). The proportions of peripheral CD4^+^ and CD8^+ ^T lymphocytes were assessed based on flow cytometry simultaneously.

**Results:**

Patients with POF showed a significantly higher value of IL-1β and hs-CRP on day 7 compared with the group of TOF (*P* < 0.05). Proportions of CD4^+ ^T cells on days 1, 3, 7 and CD4^+ ^/ CD8^+^ ratio on day 1 were statistically lower in the group of POF patients (*P* < 0.05). A CD4^+ ^T cell proportion of 30.34% on day 1 predicted POF with an area under the curve (AUC) of 0.798, a sensitivity with 61.54% and specificity with 90.00%, respectively.

**Conclusions:**

The reduction of peripheral blood CD4^+ ^T lymphocytes is associated with POF in AP, and may act as a potential predictor.

## Introduction

Acute pancreatitis (AP) is a disease characterized by local and systemic immunological response, which may result in systemic inflammatory response syndrome (SIRS), multiple organ failure and mortality [1]. With the publication of the 2012 revised Atlanta classification [2], severe AP (SAP) has been redefined as AP with POF, i.e. if OF persists more than 48h. OF that resolves within 48h is regarded as transient OF (TOF) and belongs to the group of moderately severe AP (MSAP). A recent study by Nawaz *et al*. [3] found a mortality of 15.4% among SAP patients, while no death was observed among MSAP and mild AP (MAP). Also, some other studies showed a mortality of 36–67% in patients who developed POF in the early phase of AP [2, 4–6].

At present, researchers suggest that the cytokine cascade from the innate immune system (mainly monocytes, macrophages and neutrophils) and the activated adaptive immune system (including CD4^+^, CD8^+^ T and CD19^+^ B lymphocytes) are central to the developments of SIRS, compensatory anti-inflammatory response syndrome and OF in AP [1, 7–9]. The innate immune system, and more specifically the inflammatory mediator, has been extensively explored over the past two decades. However, the activated adaptive immune system still remains to be systemically studied. T lymphocytes play a pivotal role in the regulation of the adaptive immune system, and possess a particular influence on macrophage and neutrophil activations. In a mice model of acute experimental pancreatitis, T cells, predominantly CD4^+^ subset invaded the pancreas and infiltrated border acini [10]. Pietruczuk *et al*. [11] found a significant depletion of circulating CD4^+^ T cell population in the early stage of AP, while CD8^+^ cells were in the normal range.

Alterations of the immune systems in AP patients with OF should be deeply explored given the fact that OF has become the leading cause of morbidity and mortality in these patients [4,12]. In this study, we, therefore, aimed to evaluate the role of peripheral inflammatory mediator and T lymphocyte in the progression of POF in patients with AP.

## Materials and Methods

### Patients

Between November 1^st^ 2012 and September 1^st^ 2013, a total of 69 AP patients with OF on admission presented to the Pancreatic Disease Institute of Wuhan Union Hospital were reviewed. In all patients, the time between the abdominal pain onset and admission to the hospital was within 24 hours. The diagnostic criteria were based on the presence of two or more of the following three features: 1) abdominal pain consistent with AP; 2) serum amylase and/or lipase elevation ≥ 3 times the upper limit of normal; and / or 3) computed tomography (CT) findings characteristic of AP [2]. The exclusion criteria included any of the following: age smaller than 18 years or greater than 80 years, a diagnosis of recurrent or chronic pancreatitis, a history of chronic pulmonary, renal or cardiovascular disease, pancreatitis induced by trauma or pregnant, and any surgical intervention taken in the first 3 days after admission. The study was conducted according to the principles of the Declaration of Helsinki. The need for informed consent was waived by the Medical Ethics Committee because the study was an observational one using a database from which patients’ identification information had been removed. The ethics review board of Wuhan Union Hospital approved this study.

There were 39 patients suffering from POF and 30 from TOF, respectively. OF was diagnosed according to a modified Marshall scoring system when the following cutoffs were exceeded: 1) cardiovascular failure if systolic blood pressure was < 90 mmHg with no response to fluid resuscitation; 2) respiratory failure if the ratio of PaO_2_/FiO_2_ was < 300 mmHg; and 3) renal failure if serum creatinine was **≥** 1.9 mg/dl (S1 Table). The acute physiology and chronic health evaluation II (APACHE II) scores [13] were recorded on admission and on day 1.

### Treatment

All patients received standard treatment based on the United Kingdom and Chinese Medical Association guidelines for the management of acute pancreatitis [14, 15]. Mechanical ventilation (noninvasive or invasive) and dialysis were instituted when needed. Antibiotic prophylaxis was administered for no more than 10 days, unless clinical symptoms of persistent sepsis were shown. Fluid supplementation followed international guidelines. Both crystalloids and colloids were used based on the assessment of residents. Parenteral nutrition was given, and after the function of gastrointestinal tract recovered, patients received both enteral feeding and parenteral nutrition. If a patient was confirmed of an obstruction in the common bile duct or in the ampulla of Vater by CT, an endoscopic retrograde cholangiopancreatography (ERCP) was given. CT scan was performed within 72 hours of admission before any surgical intervention. A contrast-enhanced CT was repeated after 7 to 10 days to determine the morphology of acute pancreatitis. All CT scans were reviewed by a radiologist who was blinded to the study. The presence of infected pancreatic necrosis was presumed when there was extraluminal gas in the pancreatic and / or peripancreatic tissues on contrast-enhanced CT or when percutaneous, image-guided fine needle aspiration was positive for bacteria and / or fungi on Gram stain and culture [2].

### Sample collection and measurements

#### Inflammatory mediator

Blood samples were obtained on 1st, 3rd and 7th day after hospitalization. Serum values of IL-1β, IL-6 and TNF-α were measured with enzyme-linked immunosorbant assays (ELISA) (R&D Systems, Minneapolis, Minnesota) according to the manufacturer’s instruction in Laboratory of Pancreatic Disease Institute of Wuhan Union Hospital. Serum hs-CRP value was measured by the Cobas Integra 800 (Roche, Basel, Switzerland) in Laboratory Medicine of Wuhan Union Hospital.

#### CD4^+^ and CD8^+^ T lymphocytes

Surface monocyte antigens (cluster designation, CD) of heparinized peripheral blood lymphocytes were assessed using double-stained (fluorescein isothiocyanate (FITC) / phycoerythrin (PE)) monoclonal antibodies (BD Bioscience, San Jose, CA, USA) and a flow cytometer (FACSCanto II, BD Bioscience, San Jose, CA, USA).

### Statistical Analysis

Statistical analysis was undertaken using SPSS 16.0 (SPSS Inc, Chicago, IL). Data are shown as mean ± standard deviation (SD). Group comparisons used Student’s t test for normally distributed variables and non-parametric Mann-Whitney U test for non-normally distributed variables. Categorical variables were compared with a chi-square test. Data between days 1, 3 and 7 were compared using Friedman repeated-measures ANOVA on ranks. Differences were considered to be significant at *P* < 0.05. Receiver-operating curves (ROC) were generated to determine the cut-off values for optimal sensitivity, specificity, positive and negative predictive values.

## Results

### Patient Characteristics

Baseline characteristics of the two groups are summarized in Table 1. No difference was observed regarding gender, age and etiology between POF (n = 39) and TOF (n = 30). Among the 39 POF patients, persistent solitary OF appeared in 30 (76.9%) and persistent multiple OF in 9 (23.1%). In the 30 TOF ones, 25 (83.3%) suffered from transient solitary OF and 5 (16.7%) with transient multiple OF. Twenty-three patients in the group of TOF developed acute peripancreatic fluid collection, five suffered from acute necrotic collection and two from interstitial oedematous pancreatitis. Three patients developed pancreatic pseudocyst, and one developed walled-off necrosis. There were 22 patients in the group of POF suffering from acute peripancreatic fluid collection, and 17 from acute necrotic collection. Pancreatic pseudocyst was found in 7 and walled-off necrosis in 5 of the POF ones. Infection of pancreatic necrosis was found in 3 TOF patients and in 11 POF ones, which was confirmed by positive fine needle aspiration of necrotic tissue. Infection was observed from 6 to 21 days after hospitalization, with a median time of 12 days. Twelve patients underwent pancreatic debridement, with a mean operation time of 24 days. The remaining one was treated conservatively with percutaneous drainage of organized pancreatic necrosis. No laparoscopic debridement was given. Eight patients (5 men and 3 women) died among the 39 POF patients due to infected necrosis during hospitalization, with a mortality rate of 20.5%. No death was observed in the 30 TOF ones (Table 1).

**Table 1:** Baseline characteristics of the AP patients.

	POF (n = 39)	TOF (n = 30)	*P*-value
Gender (male/female)	23 / 16	19 / 11	0.659
Age (years)	48.92 ± 11.17	47.90 ± 10.39	0.735
Etiology, n (%)			0.564
Biliary	10 (25.6%)	8 (26.7%)	
Hyperlipidemia	9 (23.1%)	9 (30.0%)	
Acohol	8 (20.5%)	6 (20.0%)	
Idiopathic	12 (30.7%)	7 (23.3%)	
Solitary organ failure, n			
Pulmonary	23	13	
Renal	6	9	
Cardiovascular	1	3	
Multiple organ failure, n			
Pulmonary + Renal	6	3	
Pulmonary + Cardiovascular	2	1	
Renal + Cardiovascular	0	1	
Pulmonary + Renal + Cardiovascular	1	0	
APACHE II score on admission	11.56 ± 4.15	8.50 ± 1.15	0.002
APACHE II score on day 1	10.95 ± 3.85	8.80 ± 1.36	0.019
Pancreatic necrosis	17	4	0.046
Mortality	8	0	0.029

Data are presented in either means and standard deviations or frequencies and percentages.

### Inflammatory mediator

**Fig 1:**
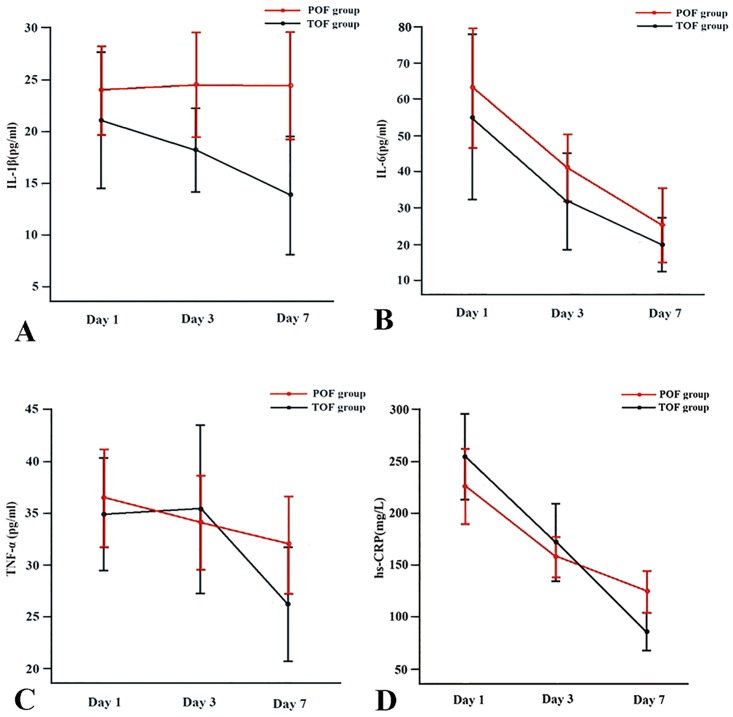
Sequential changes in the values of IL-1β (A), IL-6 (B), TNF-α (C) and hs-CRP (D) in the POF and TOF groups.

Serum peak value of IL-6 was observed on day 1, and then decreased rapidly on days 3 and 7 in both POF patients (63.08 ± 48.02 pg/ml vs. 41.10 ± 27.34 pg/ml and 25.24 ± 30.28 pg/ml, *P* < 0.01) and TOF ones (55.14 ± 48.62 pg/ml vs. 31.84 ± 28.49 pg/ml and 19.94 ± 15.90 pg/ml, *P* < 0.05). Similarly, serum level of hs-CRP peaked on day 1 and significant reductions were noticed on days 3 and 7 in group of POF (225.74 ± 105.63 mg/L vs. 157.44 ± 55.72 mg/L and 124.06 ± 55.99 mg/L, *P* < 0.01) and in group of TOF (254.37 ± 88.43 mg/L vs. 171.71 ± 77.56 mg/L and 85.99 ± 38.95 mg/L, *P* < 0.01). Serum IL-1β and TNF-α showed no statistical significance on days 1, 3 and 7 in both two groups. Values of IL-1β and hs-CRP were significantly lower in the TOF group on day 7 compared with the POF group, while values of IL-6 and TNF-α showed no significant difference between the two groups on all three days (Fig 1).

### CD4^+^ and CD8^+^ T lymphocytes

**Fig 2:**
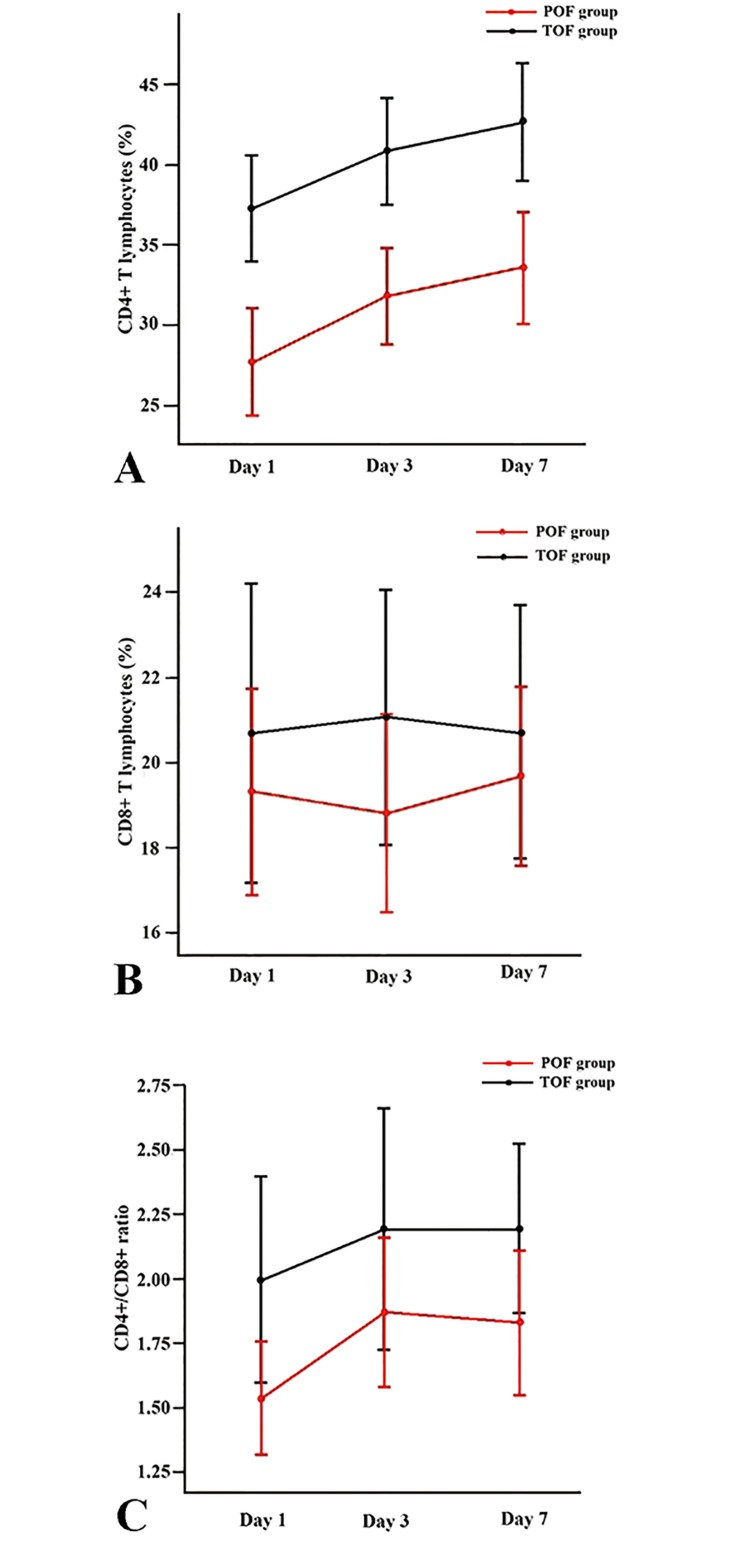
Sequential changes in the proportion of CD4^+^ T lymphocytes (A), the proportion of CD8^+^ T lymphocytes (B) and CD4^+^ / CD8^+^ ratio (C) in the POF and TOF groups. Data of CD4^**+**^ and CD8^**+**^ T cells were available in 26, 29 and 30 POF patients on days 1, 3 and 7, respectively.

Proportions of CD4^+^ T lymphocytes on days 1, 3 and 7 were significantly decreased in POF patients compared with TOF patients (27.73 ± 8.25% vs. 37.26 ± 7.04%, 31.81 ± 7.87% vs. 40.79 ± 7.07% and 33.55 ± 9.31% vs. 42.61 ± 7.81%, *P* < 0.0001). Proportions of CD8^+^ T cells were lower in the group of POF than in the group of TOF on days 1, 3 and 7, but the differences were not statistically significant. A significant reduction of the CD4^+^ / CD8^+^ ratio on day 1 was noticed in POF patients compared with TOF ones (1.54 ± 0.54 vs. 2.00 ± 0.85, *P* < 0.05), but not on day 3 and day 7 (Fig 2).

**Fig 3:**
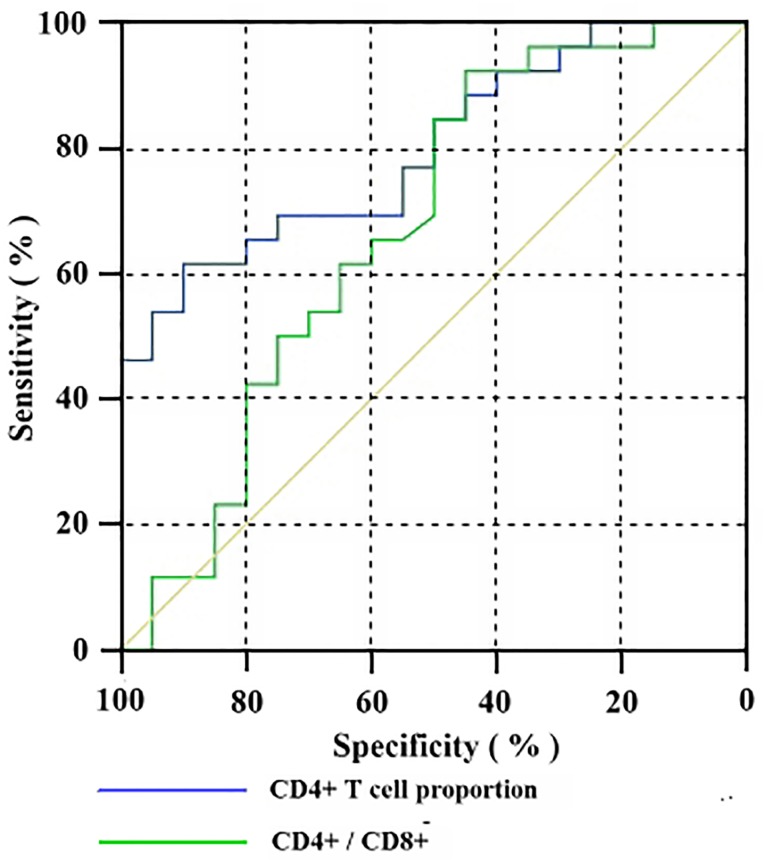
ROC curve of CD4^+^ T cell proportion versus CD4^+^/CD8^+^ ratio in predicting POF.

To determine early predictors of POF, we optimized the sensitivity and specificity, and calculated the optimal cutoff values for the proportion of CD4^+^ T lymphocytes and for the CD4^+^ / CD8^+^ ratio on day 1 using ROC curves. CD4^+^ T lymphocyte proportion on day 1 presented an area under the curve (AUC) of 0.798, with a sensitivity of 61.54% and specificity of 90.00%. The positive predictive value (PPV) was 88.89%, and the negative predictive value (NPV) was 64.29%. The optimal threshold was 30.34%. However, the CD4^+^ / CD8^+^ ratio on day 1 showed an AUC of 0.670, with an optimal threshold of 2.10 (Table 2; Fig 3).

**Table 2:** ROC analysis of CD4^+^ T cell proportion, CD4^+^ / CD8^+^ ratio and APACHE II scores in diagnosing POF.

	AUC (95%CI)	Cut—off value	Sensitivity (%)	Specificity (%)	PPV (%)	NPV (%)
CD4^+^ T cell proportion on day 1	0.798 (0.673–0.923)	< 30.34%	61.54	90.00	88.89	64.29
CD4^+^ / CD8^+^ ratio on day 1	0.670 (0.504–0.836)	< 2.10	92.31	45.00	68.57	81.82
APACHE II score on admission	0.744 (0.622–0.867)	≥ 9.5	58.97	90.00	92.00	52.94
APACHE II score on day 1	0.704 (0.574–0.834)	≥ 9.5	64.10	75.00	83.33	51.72

AUC: area under the curve; CI: confidence intervals; PPV: positive predictive value; NPV: negative predictive value.

### APACHE II score

**Fig 4:**
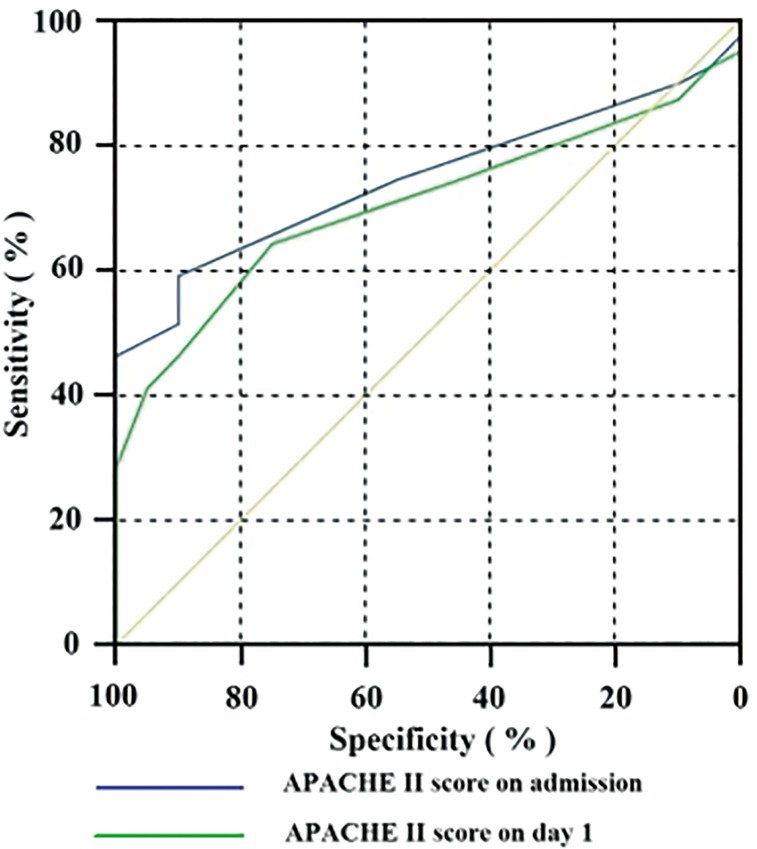
ROC curve of APACHE II score on admission versus APACHE II score on day 1 in predicting POF.

The APACHE II score on admission was 11.56 ± 4.15 in POF patients, significantly higher than those in TOF patients (8.50 ± 1.15, *P* < 0.01). A significant difference was also observed on day 1 (POF: 10.95 ± 3.85 vs. TOF: 8.80 ± 1.36, *P* < 0.05) as well. ROC analysis was performed for the APACHE II scores on admission and on day 1 (Table 2; Fig 4).

## Discussion

Studies show that 37–76% of AP patients develop OF within 24h after symptom onset [16]. POF leads to almost all deaths in the early phase of AP. It is well accepted that patients suffering from SIRS have a high risk of developing OF [1]. As a part of the host response, systemic inflammation is activated by both innate and adaptive immune systems. The former is directed by monocytes, macrophages and neutrophils, while the later is mainly made up of CD4^+^ T, CD8^+^ T and CD19^+^ B lymphocytes. Pancreatic inflammation triggers the innate immune system, and induces a cytokine cascade. However, the adaptive immune system is activated by antigens and cytokines. Exaggerated and uncontrollable host response leads to SIRS, and eventually progresses into OF [3, 4, 17].

The reductions of peripheral blood T cell subpopulations in the course of AP have been reported previously. Curley *et al*. [18] noticed a significant decrease in the proportion of CD4^+^ T cells in AP with local complications (necrosis, abscess or pseudocyst) compared with the mild form. Pezzilli and colleagues [19] observed a significant decrease of both CD4^+^ and CD8^+^ T cells in severe cases, whose severity was assessed by the clinical outcome and the number of complications developed during hospitalization. Uehara *et al*. [20] reported that CD4^+^ T cells were further decreased in the severe form compared with the mild form, while the CD8^+^ cells and the CD4^+^ / CD8^+^ ratio were not different. In that study, the severity was determined by the Ranson’s score, the APACHE II score and the Japanese 1990 criteria. Pietruczuk *et al*. [11] observed a significant decrease of the CD4^+^ T lymphocyte population in AP patients, as diagnosed according to the Ranson’s score, the Balthazar’s criteria and serum CRP levels, while CD8^+^ T cells remained in the normal range.

In this study, we focused on the relationship between T lymphocyte subgroups and POF, since POF is the main cause of early mortality in AP. Significant reduction of peripheral CD4^+^ cells in AP patients with POF was observed in a week-long period after admission compared with TOF patients, indicating that sustained lower levels of CD4^+^ T cells may be related to the progress of POF and to poor outcomes.

At present, the reasons for the reduction of peripheral CD4^+^ T lymphocytes in AP remain unclear. One of the possible reasons is the migration of activated CD4^+^ cells to the inflammatory sites, including the pancreas and other tissues like the lung and kidney [21]. In an experimental model, CD4^+^ T cell—depleted mice and nude mice (T cell—deficient) developed less severe pancreatitis than immunocompetent mice; after adaptive CD4^+^ lymphocytes transfer, this effect was partially reversed [10]. The decrease in peripheral blood CD4^+^ T lymphocytes may indicate activated CD4^+^ cells being recruited to vital organs. Another possible explanation is an excessive elimination of lymphocytes by apoptosis [11, 22], but the precise mechanism needs further research.

Early prediction of POF contributes to the appropriate management of AP patients, thus decreases morbidity and mortality. To the best of our knowledge, it is the first time that the peripheral CD4^+^ T lymphocyte proportion and the CD4^+^ / CD8^+^ ratio could be used as early predictive factors for POF in AP patients. At a cutoff value of 30.34% of CD4^+^ T cells, POF was predicted with a specificity of 90.00%.

TNF-α and IL-1β are pro-inflammatory cytokines. They are mainly released by macrophages, leading to an enhanced capillary permeability by activating neutrophils and vascular endothelia, and inducing the release of other cytokines. Studies revealed that these two mediators play a fundamental role in the progression of OF as well as pancreatic necrosis [23–26]. IL-6 is produced by monocytes and fibroblasts, and acts as the main inducer of CRP synthesis in the liver [27]. Except the immune system, IL-6 and IL-1β can be produced by multiple organs, indicating their major roles in the pathophysiology of OF [28]. There is now concordant evidence that plasma level of IL-6 correlates with the severity of AP according to the 1992 criteria [29].

In the present study, no significant differences in respect to serum values of IL-6 and TNF-α on days 1, 3 and 7 were observed between POF and TOF, suggesting that they may not play an important role in the progression of POF. On day 7, IL-1β and hs-CRP levels were significantly higher in the POF group, indicating that persistent high levels of IL-1β and hs-CRP are associated with POF. However, neither of them can be used as an early predictor.

Previously, the APACHE II score system was used to estimate the need of intensive care support for critically ill patients and to distinguish SAP from the mild form. According to the 1992 Atlanta criteria, this system may be used to determine the severity at any time during the course of AP, and the severe category was defined as a score ≥ 8 [2]. However, another study [30] classified patients as SAP if they scored > 6. Johnson *et al*. [4] observed that a fatal outcome was associated with an APACHE II score > 8. In the present study, the APACHE II scores were significantly different between the POF and TOF groups at admission and on day 1, but they showed weaker predictive abilities compared with the proportion of CD4^+^ T lymphocytes on day 1. Therefore, the proportion of CD4^+^ T lymphocytes on day 1 is comparable to the APACHE II score in the prediction of POF.

Our study had several limitations. First, as this was a derivation study the true value of CD4^+^ T lymphocytes in predicting POF remained to be investigated further in a prospective study. Second, race and ethnicity might have important bias on T cell subsets. As a result, researches including both Asian Chinese and other populations were needed. Moreover, the number of patients in our study was limited (n = 69), and we were not able to include MSAP patients without OF, as well as MAP and healthy controls. The retrospective nature did not allow us to evaluate peripheral blood lymphocyte count and anti-inflammatory cytokine IL-10 in our patients. Besides, parameters after clinical and laboratory remission were not assessed.

## Conclusions

The present study revealed that the reduction of CD4^+^ T lymphocytes in the early phase of AP is related to the progression of POF. Decreased CD4^+^ T cells may act as a potential predictor of POF in AP. The mechanism underlying needs to be further clarified in a large prospective multicenter study.

## Supporting Information

S1 Table Modified Marshall scoring system for organ failure. (DOC)

S1 Dataset Relevant data underlying the findings described in manuscript. (XLS)
