# Supplementary material for: The Reduction of Peripheral Blood CD4+ T Cell Indicates Persistent Organ Failure in Acute Pancreatitis
Source: PLoS One. 2015 May 4;10(5):e0125529. doi: 10.1371/journal.pone.0125529 (PMC4418709; doi:10.1371/journal.pone.0125529)
Supplement: S1 Table — (DOC) [file pone.0125529.s001.doc]

**S1 Table.** **Modified Marshall scoring system for organ failure**

|  | **Score** | | | | |
| --- | --- | --- | --- | --- | --- |
| **Organ system** | **0** | **1** | **2** | **3** | **4** |
| Respiratory (PaO_2_/FiO_2_) | >400 | 301–400 | 201–300 | 101–200 | ≤101 |
| Renal* |  |  |  |  |  |
| (serum creatinine, umol/l) | ≤134 | 134–169 | 170–310 | 311–439 | >439 |
| (serum creatinine, mg/dl) | <1.4 | 1.4–1.8 | 1.9–3.6 | 3.6–4.9 | >4.9 |
| Cardiovascular (systolic blood pressure, mm Hg)† | >90 | <90,fluid responsive | <90, not fluid responsive | <90, pH<7.3 | <90, pH<7.2 |

**For non-ventilated patients, the FiO2 can be estimated from below:**

| **Supplemental oxygen (l/min)** | **FiO_2_(%)** |  |  |  |  |
| --- | --- | --- | --- | --- | --- |
| Room air | 21 |  |  |  |  |
| 2 | 25 |  |  |  |  |
| 4 | 30 |  |  |  |  |
| 6-8 | 40 |  |  |  |  |
| 9-10 | 50 |  |  |  |  |

**A score of 2 or more in any system defines the presence of organ failure.**

***A score for patients with pre-existing chronic renal failure depends on the extent of further deterioration of baseline renal function. No formal correction exists for a baseline serum creatinine≥134μmol/l or≥1.4 mg/dl.**

**†Off inotropic support.**
